# Surgical treatment for anomalous origin of the right coronary artery from the pulmonary artery: a case report with five-year follow-up

**DOI:** 10.1186/s13019-020-01374-x

**Published:** 2021-01-06

**Authors:** Peng Teng, Weidong Li, Yiming Ni

**Affiliations:** Department of Cardiovascular Surgery, the First Affiliated Hospital, College of Medicine, Zhejiang University, Postal Address: 79#, Qingchun Road, Hangzhou, 310000 Zhejiang China

**Keywords:** ARCAPA, Coronary anomaly, Anomalous origin, Right coronary artery, Congenital heart disease

## Abstract

**Background:**

Anomalous origin of the right coronary artery from the pulmonary artery (ARCAPA) is a rare congenital heart disease affecting about 0.002% of the population. Knowledge of ARCAPA is almost collected from case reports. The aim of this study was to provide a rare case to better understand this rare congenital coronary anomaly.

**Case presentation:**

We report a rare case of an 18-year-old male who was initially referred because of heart murmur. Dilated and tortuous coronary arteries were detected by echocardiography and congenital coronary anomaly was suggested. Further coronary CT angiography confirmed the diagnosis of ARCAPA. Although dual coronary system provides favorable long-term outcome, bypass surgery was considered technically difficult due to the huge mismatch of caliber between the right coronary artery and graft vessels. Eventually, simple right coronary artery ligation was performed. The patient was followed up for about 5 years without evidence of atherosclerosis or myocardial ischemia.

**Conclusions:**

ARCAPA presents as a rare congenital heart disease with variable clinical manifestations. Surgical treatment is highly recommended to re-establish dual coronary system and prevent further complications. To our best knowledge, only about 200 cases of ARCAPA has been reported.

## Introduction

Anomalous origin of coronary arteries are rare which are found in approximately 1–2% of the general population [1]. Anomalous origin of the right coronary artery from the pulmonary artery (ARCAPA) was first described by the Irish anatomist John Brooks in 1885 [2]. Now it is considered as the second most common type in the coronary anomalies of pulmonary artery origination, second to the anomalous origin of the left coronary artery from the pulmonary artery (ALCAPA). In contrast to ALCAPA with high mortality rate early in childhood, the clinical manifestations in patients with ARCAPA are variable. Surgical intervention is recommended even in asymptomatic patients. Herein, we report an 18-year-old asymptomatic patient with ARCAPA who received surgery and was followed for 5 years. To our best knowledge, only about 200 cases about ARCAPA have been reported so far. Moreover, only a few cases provided such long-term follow-up data.

## Case report

**Fig. 1:**
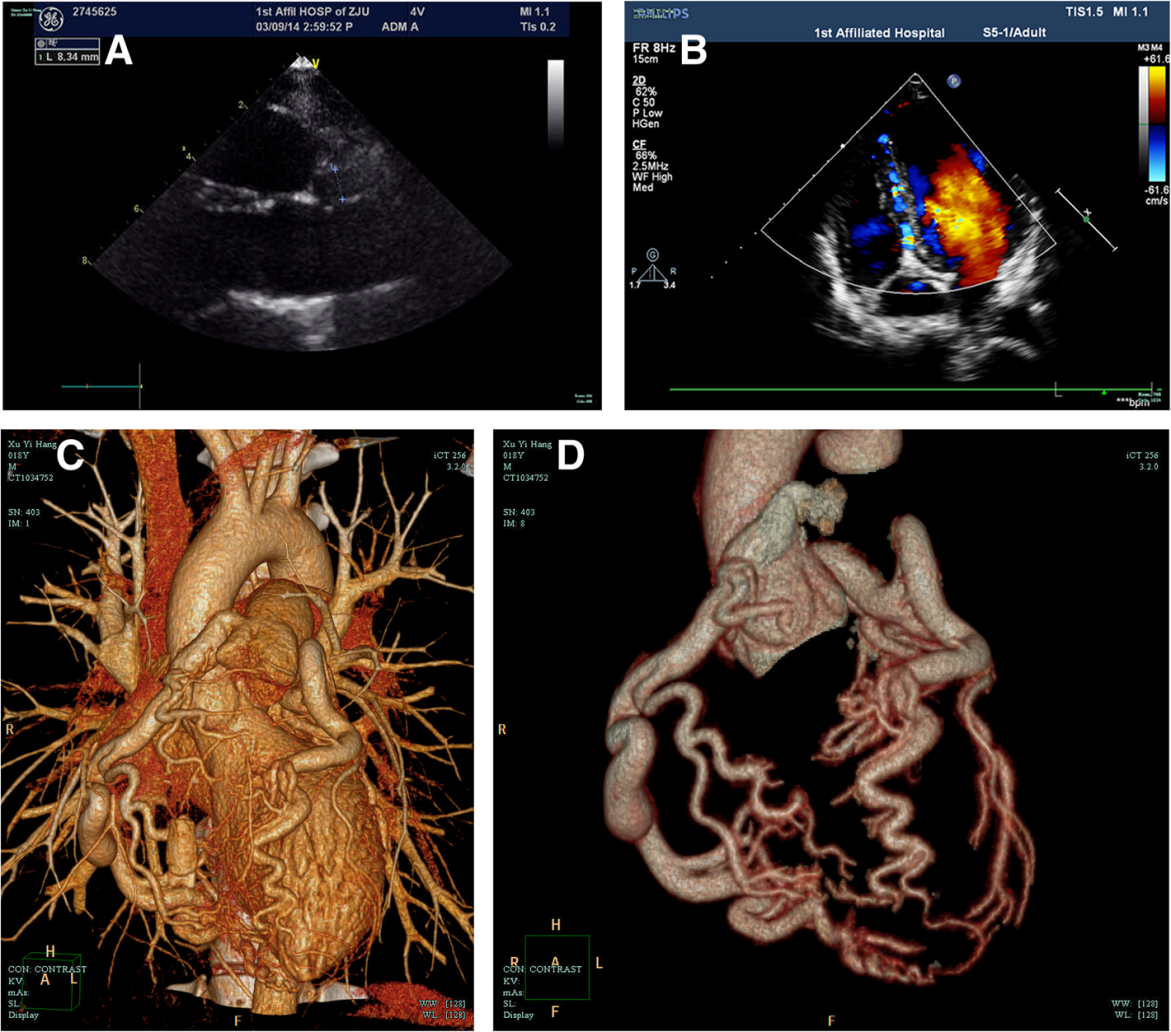
**a** TTE showed dilated ostium of RCA (white arrows) of about 8.3 mm in diameter; **b** Doppler echocardiography showed extensive collateralization with continuous flow signals within ventricular septum; **c**-**e** Coronary CT angiography showed RCA (white arrows) arose from the pulmonary artery sharing extensive collateralization with LCA (white arrowheads)

**Fig. 2:**
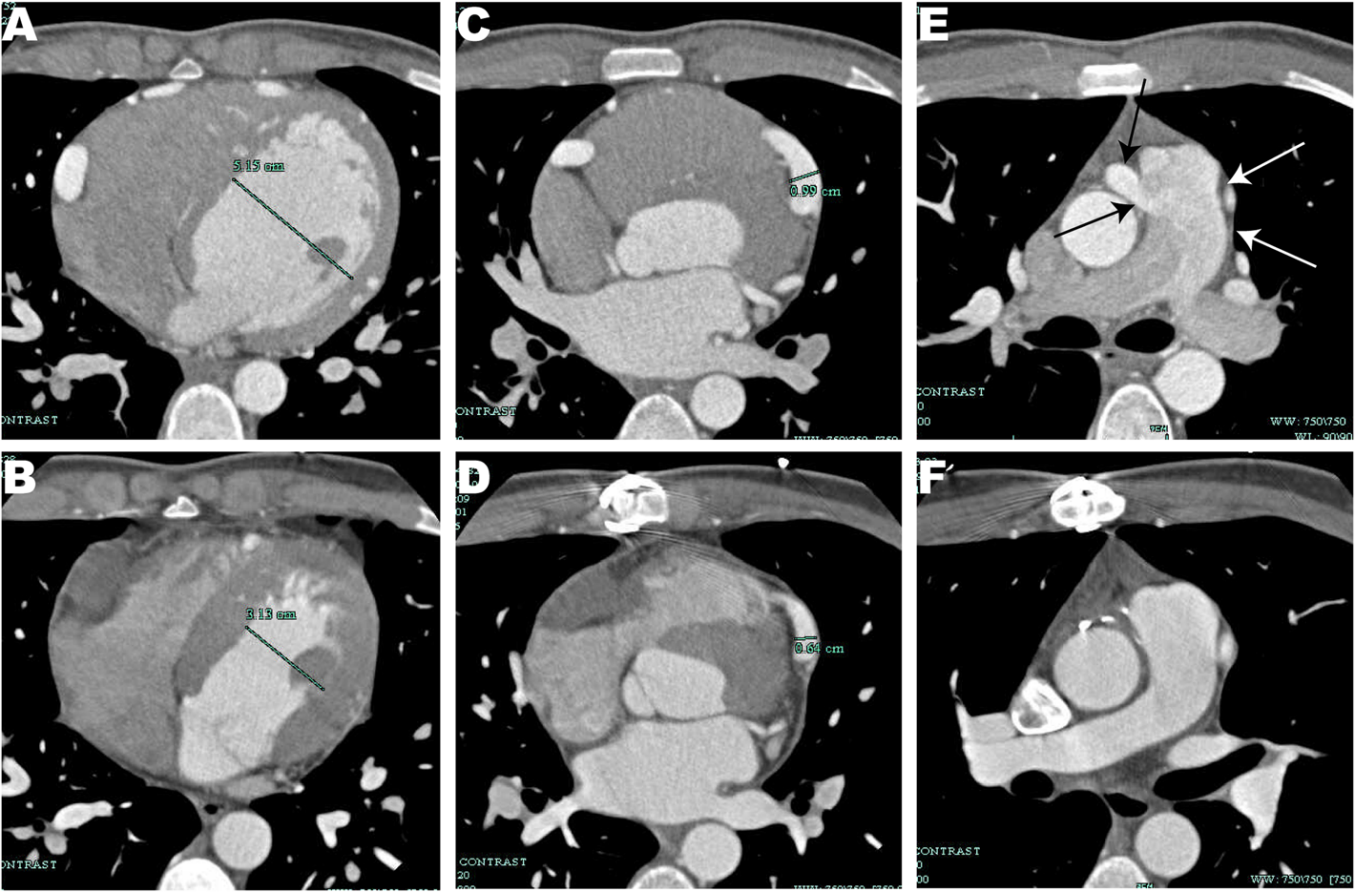
**a**, **c**, **e** Preoperative coronary CT angiography showed RCA arose from the pulmonary artery, causing increased left ventricle size (53 mm), dilated LCA (proximal left anterior descending artery of 10 mm in diameter); **b**, **d**, **f** 5-year postoperative coronary CT angiography showed complete RCA ligation with decreased left ventricle size (31 mm) and less dilated LCA (proximal left anterior descending artery of 6 mm in diameter). CT, computed tomography; RCA, right coronary artery; LCA, left coronary artery

An 18-year-old male, without previous medical history or family history of heart disease, was referred to our department due to the incidental finding of heart murmur during routine check. He was totally asymptomatic. Physical examination revealed a grade II-III/VI continuous heart murmur over his left upper sternal border. Transthoracic echocardiography showed dilated ostia of left coronary artery (LCA) and right coronary artery (RCA) with diameter of 8.8 and 8.3 mm, respectively (Fig. 1a). Coronary arteries were found diffusely dilated and tortuous with extensive collateralization within ventricular septum and apex (Fig. 1b). Additionally, the left ventricle was slightly increased (Fig. 2a). Coronary CT angiography revealed dilated and tortuous RCA, about 10 mm in diameter at proximal segment and 15 mm in diameter at its widest part, arose from the main pulmonary artery (Fig. 1c&d, Fig. 2e). Laboratory tests were unremarkable and electrocardiogram showed no evidence of myocardial ischemia. The patient was finally diagnosed as ARCAPA.

The patient was scheduled for RCA ligation with coronary artery bypass grafting (CABG). Intraoperatively, coronary arteries were found dilated, tortuous and with thrill on palpitation. RCA arose from the main pulmonary artery and shared collateralization with left coronary system which was consistent with preoperative diagnosis (Fig. 1e). Because of the huge mismatch between the luminal caliber of RCA (10–15 mm) and internal mammary artery (2–3 mm), CABG was considered technically difficult. Simple RCA ligation was assessed whether it was suitable.

**Fig. 3:**
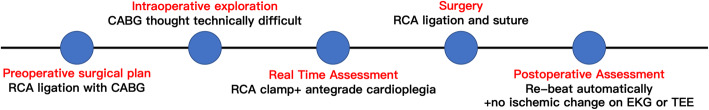
A line diagram of the diagnosis and therapy

The proximal segment of RCA was then exposed. No obvious changes were observed neither on electrocardiogram or cardiac contractility after RCA was temporarily clamped. Cardiac arrest was achieved by the antegrade perfusion of cold cardioplegia via cannula at the aortic root. During perfusion, the whole course of RCA was fully filled and the heart was completely arrest. Due to the sufficient collateralization and satisfactory cardiac arrest, simple RCA ligation was considered acceptable. The proximal segment of RCA was cut off and both stumps were closed by continuous suture with 5–0 prolene. After de-clamped the aorta, the heart re-beat automatically without obvious ischemic changes on electrocardiogram or abnormal segmental wall motion on transesophageal echocardiography. A line diagram of the diagnosis and therapy is presented in Fig. 3. The patient had an uneventful recovery except for a transient increased serum troponin (1.782 ng/ml). The patient had been followed up for about 5 years. No evidence of myocardial ischemia was detected. A 5-year postoperative coronary CT angiography showed the left ventricle as well as whole coronary system decreased in size: the left ventricle size decreased from 52 to 31 mm (Fig. 2a&b), the ostium of LCA decreased from 9 to 6 mm, the proximal left anterior descending artery decreased from 10 to 6 mm (Fig. 2c&d). No abnormal vessel was detected arising from the pulmonary artery (Fig. 2f).

## Discussion

ARCAPA was first described by the Irish anatomist John Brooks in 1885 [2]. The incidence of ARCAPA in patients undergoing coronary angiography was found to be 0.002% [3]. The embryological basis of ARCAPA remains unclear while it is generally thought with malformation of coronary artery development during the fourth to sixth week of gestation [4]. Aortopulmonary window is the most common cardiac lesions associated with ARCAPA, accounting for about one forth cases [5].

The patients with ARCAPA present variable clinical manifestations. According to previous review, about 38% of patients with ARCAPA were asymptomatic at the time of diagnosis. There is a bimodal distribution of the age at presentation as one peak centered near after birth and another peak centered around 40 to 60 years of age [5]. The reason for such phenomenon could be explained by the pathophysiology of ARCAPA. Soon after birth in patients with ARCAPA, the high pulmonary pressure keeps antegrade perfusion with deoxygenated blood. Myocardial anoxia of RCA territory causes collateralization development between the left and right coronary systems. As pulmonary pressure decreases and collateralization develops, blood from left coronary system flows retrogradely into the pulmonary artery and results in “coronary steal” phenomenon [6]. If collateralization has not sufficiently developed, the patients may present with symptoms of myocardial ischemia, which could explain the first peak of symptomatic patients near birth. Otherwise, left coronary system supplies adequate oxygenated blood to the entire heart and the patients survive and may grow up asymptomatically. However, single coronary system is more prone to atherosclerosis and left to right shunt is more prone to cardiac dysfunction which contribute to the second peak of symptomatic patients around 40 to 60 years of age.

Angiography is considered as the golden standard for the diagnosis of ARCAPA. Typical features of ARCAPA observed by angiography include retrograde flow though the RCA, extensive collateralization, dilated coronary system with increased flow as well as flow from RCA into pulmonary artery [7]. Additionally, echocardiography and coronary CT angiography are of important diagnostic value because of improved technologies and their non-invasiveness. In our case, echocardiography showed the typical features of ARCAPA including dilated coronary arteries, extensive collateralization and increased coronary flow, which were finally confirmed by coronary CT angiography.

There is consensus about early intervention in patients with ARCAPA to prevent later complications such as cardiac dysfunction and myocardial ischemia. The aims of treatment are to eliminate the “coronary steal” and establish dual coronary system originating from the aorta [8]. Three surgical strategies are commonly applied which are simple RCA ligation, RCA ligation with CABG and RCA reimplantation onto aorta. Simple RCA ligation usually took place in patients who were not deemed good candidates for CABG. The other two strategies could achieve both aims while RCA reimplantation is thought to provide higher patency rate compared with RCA ligation with CABG, although large long-term data are lacking [9]. In our case, we had planned RCA ligation with CABG preoperatively. However, the huge mismatch between the luminal caliber of RCA and graft vessels made CABG technically difficult. Because of the complete cardiac arrest and sufficient collateralization, simple RCA ligation was thought acceptable without compromised myocardial perfusion. After eliminating the “coronary steal” phenomenon, the patient received good results as 5-year postoperative coronary CT angiography showed both left ventricle and coronary system decreased in size without evidence of myocardial ischemia.

## Conclusion

We experienced a rare case of ARCAPA which was finally treated by simple RCA ligation. As patients with ARCAPA could be asymptomatic, we emphasize the suspicion in patients with dilated coronary arteries and retrograde flow in RCA when detected by echocardiography. Early intervention is necessary even in asymptomatic patients and RCA ligation with CABG or RCA reimplantation are preferred in such patients as they provide favorable long-term outcome.

## Data Availability

Please contact author for data requests.

## Abbreviations

ARCAPA: Anomalous origin of the right coronary artery from the pulmonary artery
ALCAPA: Anomalous origin of the light coronary artery from the pulmonary artery
CT: Computed tomography
RCA: Right coronary artery
LCA: Left main coronary artery
CABG: Coronary artery bypass grafting
